# Styrylpyrones from *Phellinus linteus* Mycelia Alleviate Non-Alcoholic Fatty Liver by Modulating Lipid and Glucose Metabolic Homeostasis in High-Fat and High-Fructose Diet-Fed Mice

**DOI:** 10.3390/antiox11050898

**Published:** 2022-04-30

**Authors:** Chun-Hung Chiu, Chun-Chao Chang, Jia-Jing Lin, Chin-Chu Chen, Charng-Cherng Chyau, Robert Y. Peng

**Affiliations:** 1 Research Institute of Biotechnology, Hungkuang University, Shalu District, Taichung City 43302, Taiwan; chchiu@hk.edu.tw (C.-H.C.); n404user@gmail.com (J.-J.L.); 2 Department of Program in Animal Healthcare, Hungkuang University, Shalu District, Taichung City 43302, Taiwan; 3 Division of Gastroenterology and Hepatology, Department of Internal Medicine, Taipei Medical University Hospital, Taipei 11031, Taiwan; chunchao@tmu.edu.tw; 4 Division of Gastroenterology and Hepatology, Department of Internal Medicine, School of Medicine, College of Medicine, Taipei Medical University, Taipei 11031, Taiwan; 5 Biotech Research Institute, GrapeKing Bio Ltd., Taoyuan 32542, Taiwan; gkbioeng@grapeking.com.tw; 6 Graduate Institute of Clinical Medicine, College of Medicine, Taipei Medical University, Taipei 11031, Taiwan

**Keywords:** NAFLD, *Phellinus linteus*, styrylpyrone polyphenolics, hispidin, hypholomine B, centrifugal partition chromatography (CPC), hepatoprotection, dyslipidemia, mice

## Abstract

*Phellinus linteus* (PL), an edible and medicinal mushroom containing a diversity of styrylpyrone-type polyphenols, has been shown to have a broad spectrum of bioactivities. In this study, the submerged liquid culture in a 1600-L working volume of fermentor was used for the large-scale production of PL mycelia. Whether PL mycelia extract is effective against nonalcoholic fatty liver disease (NAFLD) is still unclear. In the high fat/high fructose diet (HFD)-induced NAFLD C57BL/6 mice study, the dietary supplementation of ethyl acetate fraction from PL mycelia (PL-EA) for four weeks significantly attenuated an increase in body weight, hepatic lipid accumulation and fasting glucose levels. Mechanistically, PL-EA markedly upregulated the *pgc-1*α, *sirt1* genes and *adiponectin*, downregulated *gck* and *srebp-1c*; upregulated proteins PPARγ, pAMPK, and PGC-1α, and downregulated SREBP-1 and NF-κB in the liver of HFD-fed mice. Furthermore, the major purified compounds of hispidin and hypholomine B in PL-EA significantly reduced the level of oleic and palmitic acids (O/P)-induced lipid accumulation through the inhibition of up-regulated lipogenesis and the energy-metabolism related genes, *ampk* and *pgc-1α*, in the HepG2 cells. Consequently, these findings suggest that the application of PL-EA is deserving of further investigation for treating NAFLD.

## 1. Introduction

Nonalcoholic fatty liver disease (NAFLD) is one of the most salient causes of liver disease worldwide that will likely emerge as the leading cause of end-stage liver disease in the coming decades [1]. The global prevalence of NAFLD is 25.24%, with highest prevalence in the Middle East and South America and lowest in Africa [2], in contrast to 24.13% in the USA [1,2], and 11.5% in Taiwan [3]. In NAFLD, dyslipidemia manifests as an increase in serum triglyceride and low-density lipoprotein cholesterol levels and decreased high-density lipoprotein cholesterol levels [4]. NAFLD without histological changes is associated with complications from dyslipidemia and type 2 diabetes (T2DM), while NAFLD with histological changes is classified as NASH [4]. 

The majority of the population with NAFLD have isolated steatosis (non-alcoholic fatty liver, NAFL) and a smaller proportion develop non-alcoholic steatohepatitis (NASH), with an increase in hepatic fibrosis leading progressively to cirrhosis, liver cancer, end-stage liver disease and death [5,6,7]. Overall, cardiovascular disease (CVD) may become the leading cause of death in patients with NAFLD [5].

*Phellinus linteus* (PL), known as ‘Sanghuang’ mushroom in China and Korea, and “meshimakobu” in Japan, is one of the most important groups of medicinal macrofungi which have long been used in clinical settings for the past two centuries in many Asian countries [8,9]. A representative group of medicinal fungi, including *P. linteus*, *P. igniarius*, *P. ribis*, *Inonotus obliquus* and *I. xeranticus* was shown to produce a large and diverse spectrum of styrylpyrone-type polyphenols [10]. In a previous study, two predominant active substances were isolated and identified from the culture broth of *P. linteus*. Their chemical structures were identified as hispidin and hypholomine B and helped to elucidate the neuraminidase inhibitory activity, which plays an important role in viral proliferation for the prevention of the spread of influenza infection [11]. The styrylpyrone-type polyphenols of PL, i.e., hypholomine B and hispidin, have been reported to have a significant scavenging activity against radical species in a concentration-dependent manner. In ABTS^•+^ scavenging capabilities, hypholomine B was found to be four times greater than that of Trolox, and superior to that of hispidin [12]. In addition to the antioxidant and anti-neuraminidase activities, PL also possesses diverse bioactivities, including anti-viral [13], anti-diabetic [14], and anti-dementia properties [15]; more importantly, another traditional major indication is hypoglycemic and hypolipidemic effects for preventing type 2 diabetes [16]. 

Treatments of NAFLD with multimodal interventions such as weight loss, life style modifications and possible medication have been considered as available options [17]. Although there is a growing body of literature demonstrating the hepatoprotective [18] and anti-diabetic effects of Pl [14], it is unclear whether PL from submerged liquid culture is effective in the inhibition of NAFLD in vivo. Few studies have been performed on the production of Pl and its bioactive compounds on the large industrial cultivation of *P. linteus* (>100 L fermenter). Due to the difficulty of process handling, whether the industrial scale could produce similar active components is still unclear. To delineate the protective and therapeutic effects of *P. linteus* on NAFLD, a certain amount of the active compound that was obtained experimentally was isolated from the 2000 L-airlift fermenter products by centrifugal partition chromatography (CPC), which was used in vivo and in an in vitro model to examine whether the cited risk factors of NAFLD could be alleviated. 

## 2. Materials and Methods

### 2.1. Chemicals and Equipments

Minimum Essential Medium Alpha Medium, MEM NEAA (100×), trypsin (0.25%), Fetal bovine serum, penicillin-streptomycin (10,000 units/mL penicillin and 10,000 μg/mL streptomycin) were provided by Gibco (Grand Island, NE, USA). 2′,7′-Dichlorodihydrofluorescein diacetate (DCFH-DA), 2,2′-azinobis (3-ethyl-benzothiazoline-6-sulfonic acid) (ABTS^•+^) and 2,2-diphenyl-1-picrylhydrazyl (DPPH) free radicals and Oil Red O solution 0.5% in isopropanol were purchased from Sigma-Aldrich (St. Louis, MO, USA). The protein Assay was a product of Bio-Rad (Hercules, CA, USA). Other chemicals not mentioned were provided by Merck (Darmstadt, Germany). The antibodies used included anti-AMPK alpha 1/2 (Abcam, ab131512, 62 kDa), anti-PPAR gamma antibody- ChIP grade (Abcam, ab45036, 57 kDa), anti-PGC1 alpha (Abcam, ab54481, 92 kDa), AMPKα (Cell signaling, 2532s, 62 kDa), SREBP-1(2A4) (Novus, NB600-582), SIRT-1 poly antibody (Proteintech, 13161-1-AP, 82 kDa), NF-κB p65 (C22B4) (Cell signaling, 4764s, 65 kDa), β-actin (Taiclone, tab913655, 42 kDa). High performance liquid chromatography (HPLC) was performed with a Hitachi HPLC system including an L-2130, L-2200 autosampler and an L-2400 diode array detector, and was operated using the D-2000 Elite system software. A column block heater (Jones Chromatography, Hengoed, Wales, UK) was used and controlled at 35 °C. LC-ESI-MS data were obtained with a 6420 triple quadruple LC/MS system (Agilent, Santa Clara, CA, USA) including a 1260 Infinity HPLC system (Agilent Technologies, Santa Clara, CA, USA), a MassHunter Workstation was used for data acquisition, a degasser (model G1379B), a binary gradient pump (model G1312B), an autosampler (model G1329B), a column oven (model G1316A, maintained at 35 °C) and a photodiode array detection (PDA) system (model G1315D, 210–400 nm) were also used. The analytical column used was the Waters Symmetry C18 analysis column (150 × 2 mm i.d.; 100 Å, 3.5 µm,) which was linked with a precolumn (SceurityGuard C18 (ODS), 4 × 3.0 mm i.d., Phenomenex Inc., Torrance, CA, USA). The column oven was maintained at 35 °C. Centrifugal partition chromatography (CPC) experiments were performed on an Armen fully integrated Spot Prep instrument (Armen instrument, Saint-Avé, France). The CPC instrument equipped with one 250 mL capacity column was placed on two separated rotors. Each column is composed of eight stacked disks engraved with a total of 576 twin chambers (250 mL capacity). The CPC columns were coupled with a Spot-Prep I (Armen Instrument) integrated preparative HPLC instrument equipped with a built-in two-headed quaternary gradient HPLC pump, an injector loop (10 mL), a Flash 10 DAD 600 detector (Ecom, Prague, Czech Republic), an automatic fraction collector, and was operated using Armen Glider software.

### 2.2. Cultivation of P. linteus Mycelia

The cultivation of *Phellinus linteus* ATCC 26710 was conducted at the Grape King Biotech Research Institute (LongTan, Taoyuan City, Taiwan) as in the previous report [19]. In brief, the mycelia of *P*. *linteus* were inoculated into a 2L-broth containing 1% glucose and soybean milk (Brix 2.5) (pH 4.5) and incubated at 28 °C for 8 days with continuous aeration at 1 *vvm* and agitation at 100 rpm. Then, they were transferred into a pilot scale 500 L fermenter (temperature 28 °C; aeration rate 0.5 *vvm*; agitation speed 60 rpm) and fermented for 8 days, and finally into a 2000 L fermenter (working volume 1600 L; temperature 28 °C; aeration rate 0.5 *vvm*; agitation speed 60 rpm), in which they were fermented for 12 days. The broth was centrifuged to collect the mycelia, which were lyophilized and stored at −20 °C for use. 

### 2.3. Preparation of P. linteus Mycelial Extract

The lyophilized *P. linteus* mycelia powder was extracted with pure water, with 25, 50, 75 or 100%% methanol, respectively, at a solid to solvent ratio 1:10 (*w*/*v*), were then ultrasonicated at ambient temperature for 30 min and suction filtered through a 0.45 μm PTFE membrane to collect the filtrate. The residue was similarly re-extracted twice. The filtrates were combined and evaporated under reduced pressure until dry. The reclaimed percentage was calculated, and the desiccated powder was stored at −30 °C for use. The best yield containing the highest total polyphenol content (TPC) from the extraction was then selected as the sample for the following solvent partition (hereafter denoted as PL for this extract).

### 2.4. Solvent Partition of PL

The PL was dissolved in 20 mL deionized water and ultrasonicated for 10 min. The aqueous PL solution was sequentially partitioned with n-hexane, ethyl acetate, and n-butanol, thrice by each. Each partition was separately combined and evaporated under vacuum. The desiccated powder was taken for its weight and stored at −20 °C. The ethyl acetate fraction was used for animal experiments and denoted as PL-EA hereafter. 

### 2.5. Purification of PL-EA with Centrifugal Partition Chromatography

Based on our previous report [20] concerning CPC purification methods, the obtained PL-EA products were further purified using CPC using the two-phase solvent system. In brief, petroleum ether-ethyl acetate-methanol-water solvent systems in different proportions (Supplementary Table S3) were used in the study for the purification of hispidin and hypholomine B with one-step separation.

### 2.6. HPLC and HPLC/ESI-MS-MS Analyses of PL-EA Extract

The sample injection volume was 10 μL, and the flow rate of the mobile phase was 0.3 mL/min. The mobile phase consisted of solvent A (H_2_O containing 0.1% formic acid) and solvent B (acetonitrile containing 0.1% formic acid). The gradient elution was programmed as (time in min, A:B): at 0 min, A:B = 90:10; at 3 min, A:B = 90:10; at 10 min, A:B = 65:35; at 15 min, A:B = 50:50; at 30 min, A:B = 5:95; at 40 min, A:B = 5:95; at 45 min, A:B = 90:10; and at 60 min, A:B = 90:10. After the compounds were eluted and separated, they were further identified with a triple quadrupole mass spectrometer. Nitrogen was used as the drying gas whose flow rate was 9 L/min and temperature was 300 °C. The nebulizing gas was operated at 35 psi. The other parameters were as follows: the potential, 3500 V; the fragmentor voltage, 90 V; and the collision voltage, 15 V. The quadrupole 1 filtered the calculated the m/z of compound of interest, while the quadrupole 2 scanned the ions produced from nitrogen collision with these ionized compounds in the range 100–800 m/z within a scan time of 200 ms/cycle. A multiple reaction monitoring (MRM) mode was used for the MS data acquisition of PL-EA extract. The detection parameters of target compounds and internal standard (IS, quercetin) are summarized in Supplementary Table S1 and their MRM chromatograms are shown in Supplementary Figure S1. A quantitative analysis was performed using the IS method acquired in the MRM mode which was employed for quantifying each target from the obtained peak area. Data were collected and analyzed with the Agilent MassHunter Workstation B.01.04 Software. 

### 2.7. Animal Experiment

To validate the inhibition of PL-EA on NAFLD in vivo, biochemical and histological changes regarding mice liver tissues were investigated. 

Male C57BL/6 mice were purchased at 5 weeks of age from BioLASCO Taiwan Co., Ltd. (Yi-Lan, Taiwan). These mice were authorized for admission to the University animal room by The University Ethic Committee of Animals Care and Protection (license code: 10419). In the first week, the animals were caged in the animal room and maintained at a light control of 12 h light/12 h dark cycle, the animal room was maintained at 22 ± 2 °C, in relative humidity (RH) 65 ± 5%. Mice were provided ad libitum access to water from a reverse osmosis system. During a 14-week period study, the mice were grouped into four experimental groups, the normal control (C), the high fat high fructose diet (HFD) control, the HFD + high dose PL-EA (PL-EA-H), and the HFD + low dose PL-EA (PL-EA-L). Group C was fed with regular chow, while the other three groups were given HFD. The NAFLD model was induced in mice with the HFD chow containing fat 40%, fructose 22%, and cholesterol 2% (Research diet, New Brunswick, NJ, USA) [21]. The oral glucose tolerance tests (OGTT) were carried out, respectively (1 g of glucose/kg bw) at week 9 (after induction) and week 14 (after treated). The PL-EA-H (70 mg/kg) and PL-EA-L (35 mg/kg) were administered daily by oral gavage after week 10. The whole experiment lasted for 14 weeks. The animals were kept fasted overnight, CO_2_-euthanized, and the organs were dissected immediately, rinsed with sterile saline and stored at −20 °C for further use. 

### 2.8. Oral Glucose Tolerance Test (OGTT)

The oral glucose tolerance test (OGTT) was carried out at week 9 after induction for 8 weeks, and a second time OGTT was conducted at week 14 after the administration of PL-EA for 4 weeks. In brief, after having been fasted for 12 h, the experimental mice were tail-vein bled, the blood sugar level was measured to serve the zero point reference. The mice were then fed glucose solution 0.2 mL by oral gavage, at a dose of 3 g/kg. After tube fed, the blood sugar level was tested at 30, 60, 90, and 120 min, to establish the plasma glucose concentration–time course curve, from which the result of OGTT was determined. 

At week 14, the experimental mice were fasted for 12 h and CO_2_-euthanized. After assured unconsciousness, blood was withdrawn from the heart using a 1 mL syringe with a 26 G needle and transferred into the heparin coated gel separator green tubes. The blood was centrifuged with 3500× *g* at 4 °C. The supernatant plasma was separated and subjected to biochemical tests within 24 h. Livers, kidneys, and spleens were dissected and rinsed twice with sterile saline. The adhering water was adsorbed with heavy tissues. The organs were weighed, wrapped with aluminum foil, and frozen with liquid nitrogen. The liver was divided into five sections, the left lobe was divided into two pieces, one immersed in formalin, and the other and the remaining parts were separately classified and wrapped with aluminum foil and frozen at −80 °C for use. 

### 2.9. Biochemical Measurements for Blood

The plasma was analyzed with the Fuji automatic biochemical analyzer (FUJI DRI-CHEM 3500s) for total cholesterol (T-CHO), low density lipoprotein-cholesterol (LDL-C), high density lipoprotein-cholesterol (HDL-C), triglycerides (TG), uric acid (UA), glutamic oxaloacetic transaminase (GOT; or aspartate transaminase, AST), and glutamic pyruvic transaminase (GPT; or alanine aminotransferase, ALT). 

### 2.10. Histological Examination 

The organs dissected from the mice were immersed in 10-fold volume formalin (10%) and agitated overnight to fix. The fixed tissues were rinsed under flowing tap water for 30 min, immersed in sequentially increasing concentrations of ethanol, starting from 70%, 80%, 85%, 90%, and 95%, each for 1 h, and then in absolute alcohol for 1.5 h and this was repeated thrice before finally being immersed twice in xylene. The treated tissues were immersed twice in liquid paraffin at 57 °C, each time for 2 h, to prepare the paraffin-embedded tissues and slices.

### 2.11. Hematoxylin-Eosin Staining

Liver tissues were formalin-fixed, embedded in paraffin, sectioned into 2 µm, and subjected to H&E using the conventional protocol, and the images were photographed according to the previous report [21]. 

### 2.12. Oil-Red O Staining

We adapted the method of Cui et al. [22] with a slight modification, in which the paraffin-embedded tissues were sliced with a microtome and stained with 0.3% Oil Red O solution for 1 h at ambient temperature, rinsed with deionized water and mounted onto the microscope to inspect the oil drop distribution profile. 

### 2.13. Protein Extraction and Western Blot Analysis

A previous report on the expression analysis of proteins, including PGC-1α, AMPK, p-AMPK, SREBP-1, NF-κB and PPARγ in liver tissues, was followed, although a slight modification was introduced [21]. In brief, the liver tissues were homogenized in the RIPA buffer containing protease inhibitors. An total of 30–40 µg protein was loaded and separated in a 10% SDS-PAGE and electro-blotted to the nitrocellulose membranes. After blocking with TBS buffer (20 mM Tris–HCl, 150 mM NaCl, pH 7.4) containing 5% non-fat milk, the membrane was incubated overnight at 4 °C with various specific antibodies including PGC-1α (1:1000; #ab5448), AMPK (1:1000; #ab1315120), p-AMPK (1:1000; #ab23875), PPARγ (1:500; #ab45036), NF-κB (1:1000, #ab16502) and SREBP-1 (1:5000; #ab26481) from Abcam (Cambridge, UK), and β-actin (1:3000; #MAB1501; Millipore, Billerica, MA, USA), followed by treatment with horseradish peroxidase-conjugated anti-mouse IgG. The results were visualized with the ECL chemiluminescent detection kit (PerkinElmer, Waltham, MA, USA) and quantified using the Image J gel analysis software. 

### 2.14. RNA Isolation and Quantitative Real-Time PCR (qPCR) 

RNA from hepatic tissues was isolated to quantify gene expression with RT-qPCR. Total RNA was extracted by using TRIzol^®^ reagent (ThermoFisher Scientific, Waltham, MA, USA), and 1.5 µg of total mRNA was reverse-transcribed using a Takara PrimeScript RT Reagent Kit (Takara Bio, Mountain View, CA, USA), following the instructions provided by the manufacturer. Amplification and detection were performed with the StepOnePlus™ Real-Time PCR System (Applied Biosystems, Foster City, CA, USA). The DNA fragments were amplified for 40 cycles (enzyme activation: 20 sec at 95 °C, hold; denaturation: 3 s at 95 °C; annealing: 40 s at 60 °C). The gene expression of β-actin was determined as the internal control and the relative expression level was calculated by using the standard 2^−∆∆Ct^ method. Primers sequences are listed in Supplementary Table S2.

### 2.15. HepG2 Cells Experiments

The HepG2 human hepatocellular carcinoma cell line (ATCC CRL-11997) was purchased from the Bioresources Collection and Research Center (Shin-Chu, Taiwan). HepG2 cells were cultured in a minimum essential medium (MEM) containing 10% fetal bovine serum, 1% penicillin-streptomycin, 1% sodium pyruvate, 1% non-essential amino acids and maintained in humidified 5% CO_2_/95% air at 37 °C. 

### 2.16. Fatty Acid Induced Mimic Hepatosteatosis in HepG2 Cells and the Treatment of Purified Compounds 

Oleic (O) and palmitic (P) acids, both fatty acids, were applied at a molar ratio of 2:1 to induce lipid deposition of HepG2 cells [23]. In brief, after reaching 80% confluence, the HepG2 cells were cultured with serum-free medium containing 1% fat-free bovine serum albumin (BSA) and exposed to 400 μM of fatty acids O/P (2:1) and incubated for 24 h to induce a mimic steatosis. Later, the supplementation of hispidin or hypholomine B at 10 or 50 μM, respectively, was added to the wells and plates incubated for another 24 h. 

To detect the lipid accumulation, the control and hispidin- or hypholomine B-treated HepG2 cells were fixed with 10% formalin for 30 min, and then stained with Oil Red O solution for 10 min. The cells were washed three times with physiological saline (PBS) and observed under an inverted microscope. To quantify lipid accumulation in the HepG2 cells, isopropanol was added to dissolve the Oil Red O reagent and the absorbance was measured at 500 nm.

### 2.17. Analysis for Gene Expression in HepG2 Cells

In our previous report [24] on the extraction of RNA, reverse transcription of RNA to cDNA and the quantification of gene expression using real-time PCR was followed. In brief, each of the 1.5 μg RNA isolated from the O/P-induced and hispidin or hypholomine B-treated HepG2 cell were used to synthesize cDNA. A real-time polymerase chain reaction was conducted according to the manufacturer’s instructions with the KAPA SYBR^®^ Fast one step ABI Prism^®^ (Sigma-Aldrich). The 2^−ΔΔCT^ value for each sample was analyzed with the StepOnePlus TM Real Time PCR System (Applied Biosystems, Thermo Fisher). Primer sequences are listed in Table S2.

### 2.18. Statistical Analysis

The data are presented as a mean ± SD result and further analyzed using the GraphPad Prism program (GraphPad, San Diego, CA, USA). The comparison within groups was evaluated using a one-way analysis of variance (ANOVA). Tukey’s post hoc test was further used for an analysis of the significance of differences among the means. A confidence level of *p* < 0.05 was considered to be statistically significant.

## 3. Results and Discussion

### 3.1. Yield of Extraction from Different Solvent and the Solvent Partition

The following five extraction solvents were used: pure water, 25, 50, 75 and 100% methanol. The results are given in Table 1. Although the yields from *P. linteus* mycelia using the 50% methanol extraction were slightly higher than when using the 75% methanol extract, the best results for the total polyphenol contents (TPC) were obtained using the 75% methanol extract (contents were 1.34 fold as high as those obtained with 50% methanol). The 75% methanol extract (PL) was then selected to obtain the PL extracts, and was used for the liquid–liquid solvent partition in the preparation of the polyphenol-enriched sample. As a result, the yield from the fraction of ethyl acetate (PL-EA) partition was 20.61 ± 1.16 mg/g d.w. 

### 3.2. Evaluation of the Distribution Coefficient (K_d_) for CPC

A suitable K value should be between 0.5 and 3.0 [25], from which the optimum solvent system can be selected for the purification of targets by using the centrifugal partition chromatography (CPC). The two-solvent system consisting of petroleum ether- ethyl acetate-methanol-water (1.6:2.3:1.0:2.1, *v*/*v*/*v*/*v*) was determined for the isolation and purification of hispidin and hapholomine B (Supplementary Table S3). 

### 3.3. Identification and Quantification of Constituents of PL-EA by HPLC-ESI-MS/MS

**Figure 1:**
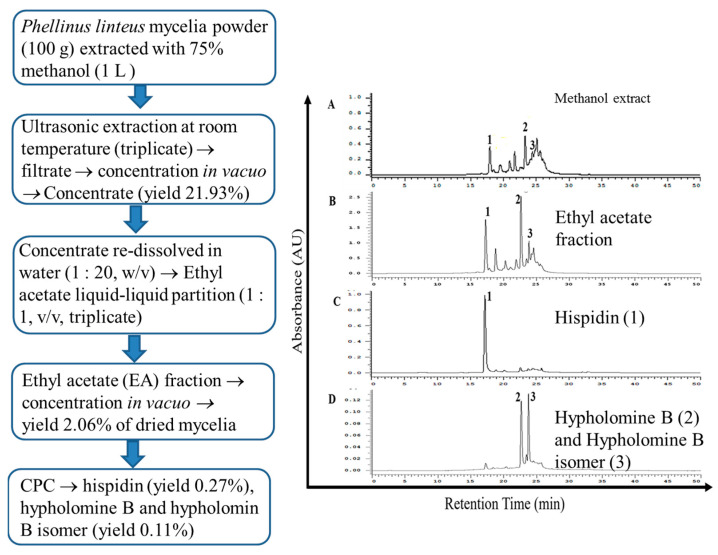
Experimental procedures of solvent extraction, partition and purification of extract prepared using the *Phelinus linteus* mycelia extract and HPLC analysis of extracts obtained in each procedure. (**A**) 75% methanol. (**B**) ethyl acetate. (**C**) hispidin. (**D**) hypholomine B and hypholomine B isomer. Peaks 1: hispidin; 2: hypholomine B, and 3: hypholomine B isomer.

The 75% methanol extract (yield of 21.93%, *w*/*w*) of *P*. *linteus* mycelia (PL) showed an HPLC profile containing hispidin (peak 1), hypholomine B (peak 2), and hypholomine B isomer (peak 3) and many other unidentified compounds (Figure 1A), which after liquid–liquid extraction with n-hexane to remove the lipids, followed by extraction with ethyl acetate (PL-EA), yielded a fraction that was abundant in antioxidants (Figure 1B) with a yield of 2.06% (*w*/*w*). These styrylpyrones were further confirmed and quantified using a HPLC-ESI(−)-MRM analysis, as shown in Supplementary Figure S1. Finally, isolation with CPC yielded purified hispidin (purity > 95%, Figure 1C), and hypholomine B and hypholomine B isomer (PL-HB) (purity > 90%, Figure 1D). As can be seen, CPC efficiently improved their purity and the majority of non-phenolic compounds were efficiently removed.

Previously, in an analysis of the ethyl acetate fraction of the 70% methanolic extract of the fruiting bodies of *Phellinus linteus*, Min et al. [26] showed the occurrence of styrylpyrone-class compounds, davallialactone, hispidin, hypholomine B, and caffeic acid [26], while Lee and Yun isolated nine compounds with ethyl acetate-soluble fractionation of *P. linteus* fruiting bodies and identified new compounds, such as protocatechuic acid, protocatechualdehyde, ellagic acid, interfungin A, and inoscavin A [10]. More recently, the same group further isolated new compounds from the culture broth of *P. linteus*, which included inotilone, 4-(3,4-dihydroxyphenyl)-3-buten-2-on, phellilane H, (2E,4E)-(+)-4′-hydroxy-γ-ionylideneacetic acid, and (2E,4E)-γ-ionylideneacetic acid [27]. 

### 3.4. The Antioxidative Capability

#### 3.4.1. DPPH Radical Scavenging Capability

**Figure 2:**
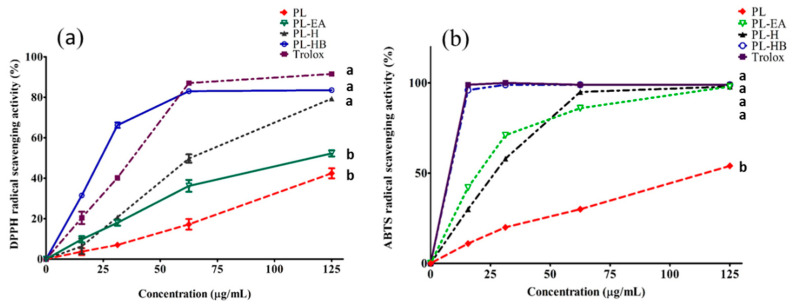
Radical scavenging capabilities of different preparations fractionated from *P. linteus mycelia.* (**a**) for DPPH radicals. (**b**) for ABTS^+^ radicals. PL: 75% methanol crude extract. PL-EA: ethyl acetate fraction. PL-H: CPC isolated hispidin. PL-HB: CPC isolated hypholomine B and hypholomine B isomer. Data are expressed as mean ± SD from triplicate experiments. Different letters in lower case on each curve indicate significantly different from each other (*p* < 0.05).

The DPPH radical scavenging capability of each partition (or isolated compound) was found to increase in a dose-dependent manner. The strongest was PL-HB (hypholomine B and its isomers) which was comparable to Trolox (Figure 2a). The order of antioxidative capabilities at 125 μg/mL was as follows: Trolox = PL-HB = PL-H > PL-EA > PL (Figure 2a), consistent with [12], the capability of hispidin oligomers to scavenge DPPH free radical was in the following order: hypholomine B > 1,1-distyrylpyrylethane > 3,14′-bihispidinyl > hispidin [12]. Chang et al. indicated that the ethyl acetate fraction exhibited strong DPPH radical-scavenging activity as well as antioxidant activities (IC_50_ = 0.66 ± 0.01 mg/mL) [28].

Hispidin exhibited quenching effects against DPPH radicals, superoxide radicals, and hydrogen peroxide in a dose-dependent manner [29,30,31], and at 1.0 mM it inhibited 85.5% of the DPPH radicals [31].

Previously, Jeon et al. isolated 10 antioxidants from the fruiting bodies of *P*. *linteus* including hispidin, davalliallactone, interfungins A, and hypholomine B, etc., and demonstrated that davalliallactone and interfungins A exhibited the strongest inhibitory effect against the DPPH radicals [32], indicating that more powerful and therapeutically useful antioxidants can be achieved if the whole fungi are utilized. 

A wide spectrum of the literature demonstrated that many species of *Phellinus* including *P. linteus*, *P. igniarius* and *P. durissimus* all showed significant DPPH scavenging capability [33,34,35], which, as suggested, can be attributed to various antioxidant compounds contained in genus Phellinus, such as caffeic acid, davalliallactone, ellagic acid, hispidin, hypholomine B, inoscavin A, interfungins A, methyldavallialactone, protocatechualdehyde and protocatechuic acid [36], which is in agreement with our findings (Figure 1B,C).

#### 3.4.2. ABTS^+^ Radical Scavenging Capability

Similar to the results found for the scavenging capability of DPPH, the anti-ABTS^+^ radical capability of different extracts from lyophilized *P*. *linteus* mycelia increased in a dose dependent fashion, and the order (in decreasing tendency) was as follows: Trolox = PL-H = PL-EA = PL-HB > PL at dose 125 μg/mL (Figure 2b) (*p* < 0.05). With regard to the anti-ABTS^+^ capability, it was positively correlated with the total phenolic contents (Figure 2b), which is in good agreement with Chang et al. [28]. In particular, caffeic acid, inotilone, and 4-(3,4-dihydroxyphenyl)-3-buten-2-one were potent ABTS^+^ scavengers which showed IC_50_ values of 0.52 ± 0.10, 1.10 ± 0.10, and 1.69 ± 0.11 μM, respectively [24]. The *P*. *linteus* ethanolic extract showed a powerful capacity in scavenging DPPH and ABTS^+^ radicals, of 2–10 fold stronger than any other species of mushrooms [26]. 

### 3.5. Body Weight Variation of Experimental Mice 

**Figure 3:**
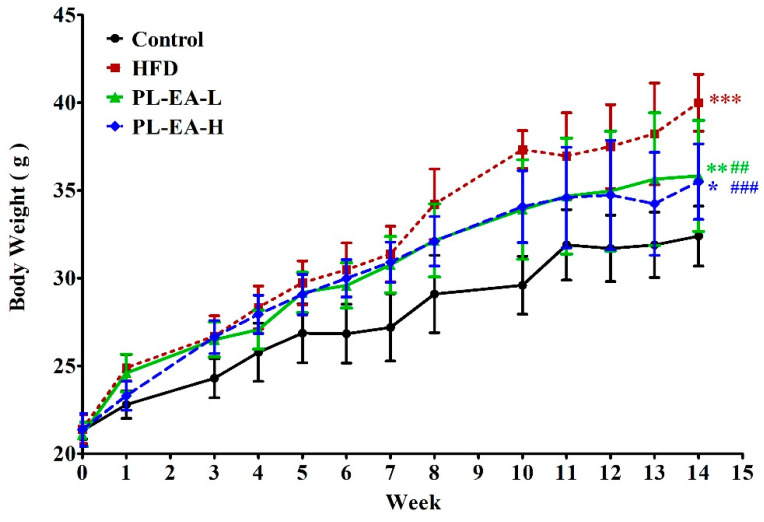
PL-EA inhibits high-fat high-fructose (HFD)-induced obese in mice. HFD: high-fat/high-fructose diet. PL-EA-H: HDF + high dose PL-EA (70 mg/kg). PL-EA-L: HDF + low dose PL-EA (35 mg/kg). Data are expressed as mean ± SE (*n* = 9). ## *p* <0.01; ### *p* < 0.001 vs. the HFD group; * *p* < 0.05; ** *p* < 0.01; *** *p* < 0.001 vs. the control.

A mouse model of obesity was successfully established by feeding them a high-fat high-fructose diet (HFD) for 10 weeks (control: 32.36 ± 1.71 g; HFD: 40.00 ± 1.62 g) (Table 2). The body weight of all experimental mice increased steadily during the whole period of the experiment within 14 weeks, while the HFD mice group consistently showed the largest body gain (Figure 3). PL-EA-H and PL-EA-L treatments did not affect the mice’s slow weight gain trend, indicating the nontoxic nature of the PL-EA fraction. More importantly, the PL-EA extract seemed to exhibit a rather promising sliming effect (Figure 3). A similar experiment conducted by Noh et al. demonstrated davalliallactone to be the active compound responsible for reducing the body weight gain [37].

### 3.6. The Ratio of Liver to Body Weight 

The liver weight in mice normally falls in the 2–3 g range (3–5%/bw) [38]. Consistent with this, we showed the liver weight and the ratio of liver to body weight (in %) in the experimental control of mice to be 1.89 ± 0.25 g or 5.83 ± 0.61%, respectively (Table 2). HFD induced a higher liver weight (3.22 ± 0.48 g), hence leading to a higher ratio (8.06 ± 1.19%) (Table 2), implicating the occurrence of fatty liver. Treatment with PL-EA dose dependently alleviated these abnormal liver weight as demonstrated by the results of 2.49 ± 0.44 and 2.31 ± 0.04 g, or in the ratio of liver to body weight 7.02 ± 0.70 and 6.48 ± 0.75% by PL-EA-L and PL-EA-H, respectively (Table 2), indicating the powerful anti-obesity effect of PL-EA. Studies in the literature emphasize the importance of the percentage of total body mass to assess the metabolic or nutritional status although it has been noted that in mice livers the results are more prominent than that of rats or humans [38]. 

### 3.7. Plasma Biochemical Measurements

#### 3.7.1. The Total Plasma Cholesterol Level

**Figure 4:**
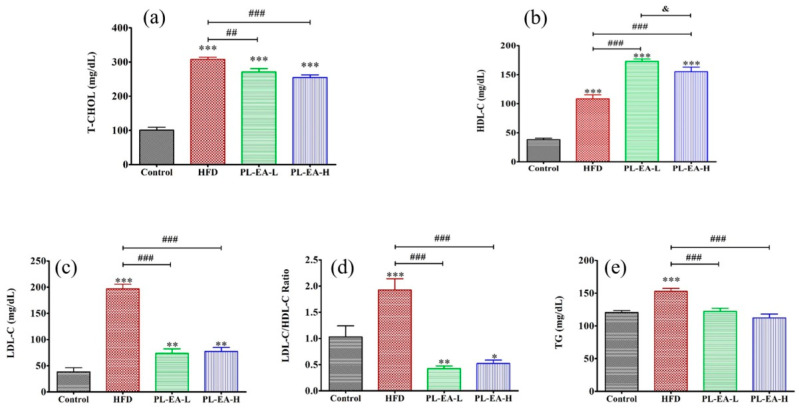
Effects of ethyl acetate fraction of *P. linteus* mycelia on the lipid profile. (**a**) total plasma cholesterol content, (**b**) content of plasma high density lipoprotein cholesterol, (**c**) content of plasma low density lipoprotein cholesterol, (**d**) the ratio LDL-C/HDL-C and (**e**) the plasma TG content. HFD: high-fat/high-fructose diet (HFD). PL-EA-L: HDF + low dose PL-EA (35 mg/kg). PL-EA-H: HDF + high dose PL-EA (70 mg/kg). Data are expressed as mean ± SE (*n* = 9). ## *p* < 0.01; ### *p* < 0.001 vs. the HFD group; * *p* < 0.05; ** *p* < 0.01; *** *p* < 0.001 vs. the control. ^&^ *p* < 0.05 significant differences between the groups.

The total plasma cholesterol (TC) level in the control, the HFD, the PL-EA-L and PL-EA-H mice lay in the following range: control (100 ± 10 mg/dL); HFD (315 ± 7 mg/dL) (*p* < 0.001), PL-EA-L (270 ± 12 mg/dL), and PL-EA-H (260 ± 8) mg/dL, respectively (*p* < 0.001) (Figure 4a), implicating that PL-EA effectively reduced the TC. Statistically, adults with high TC/HDL-C or TG/HDL-C ratios, or both, have a greater risk of NAFLD, especially advanced NAFLD [39,40].

#### 3.7.2. The Plasma HDL-C Level

The plasma HDL-C level in the control group was 37.5 ± 4.0 mg/dL, while that of HFD was elevated at 107.0 ± 1.0 mg/dL. Interestingly, the PL-EA-L, and PL-EA-H were alleviated across the board, with higher levels of HDL-C to 175.0 ± 8.0, and 156.0 ± 14.0 mg/dL, respectively (Figure 4b). Epidemiological studies have suggested an inverse correlation between high-density lipoprotein-cholesterol (HDL-C) levels and the risk of cardiovascular diseases and atherosclerosis [41]. A 1% increase in HDL-C level is associated with a 2% decrease in CV risk [42,43]. 

#### 3.7.3. The Plasma LDL-C Level

The HFD elevated the plasma LDL-C level to 193 ± 8 mg/dL compared to 40 ± 7 mg/dL in the control (*p* < 0.001). PL-EA effectively suppressed its level to 73 ± 8 and 74 ± 7 mg/dL, respectively (*p* < 0.05) (Figure 4c). Patients with higher LDL-C levels are more likely to have a higher prevalence of NAFLD than subjects with lower levels [44]. 

#### 3.7.4. Ratio of HDL-C/LDL-C

A high fat/high fructose diet induced a high ratio of LDL-C/HDL-C to 1.91 ± 0.25, compared to 1.05 ± 0.22 of the control (*p* < 0.001) (Figure 4d). Interestingly, the ethyl acetate fractions, despite the low or the high level of EA, all efficiently lowered the ratio to 0.43 ± 0.07 and 0.55 ± 0.09 (Figure 4d). 

More recently, Wang et al. suggested that the ratio of non-HDL-C to HDL-C would be a better predictor for new-onset NAFLD [45].

#### 3.7.5. The Plasma Triglyceride Levels 

The plasma triglycerides were significantly elevated in HFD mice, reaching 158 ± 6 mg/dL compared to 115 ± 5 mg/dL in the control, which was efficiently alleviated by the administration of PL-EA to 120 ± 7 mg/dL and 110 ± 8 mg/dL, respectively, at a high and low level of PL-EA (*p* < 0.001) (Figure 4e).

Elevated plasma TG levels are also often associated with low HDL-C levels [46]. Recently, non-HDL cholesterol (including LDL-C and remnant lipoproteins such as VLDL-c and IDL-C) has been proposed to be a better estimate of total atherogenic burden than LDL-C, especially in patients with elevated plasma TGs ranging between 200 and 500 mg/dL [47,48]. 

Hispidin was demonstrated to decrease the intracellular triglyceride content by 79.5 ± 1.37%, stimulate glycerol release by 276.4 ± 0.8% and inhibit lipid accumulation by 47.8 ± 0.16% [49]. Hispidin also inhibited glycerol-3-phosphate dehydrogenase (GPDH) and pancreatic lipase, representing the most potent inhibitors [49]. A similar experiment conducted by Noh et al. demonstrated davallialactone to be the active compound responsible for reducing hepatic lipid concentrations, and fat accumulation in epididymal adipocytes [37]. The mechanism of which was proposed to be partly mediated by the inhibition of enzymes associated with hepatic and intestinal lipid absorption and synthesis [37]. Suggestively, the presence of davallialactone in our ethyl acetate fraction (not shown), as previously reported elsewhere [10,26], might also synergistically contribute to the lowering of TG. 

#### 3.7.6. Plasma Level of GOT and GPT

**Figure 5:**
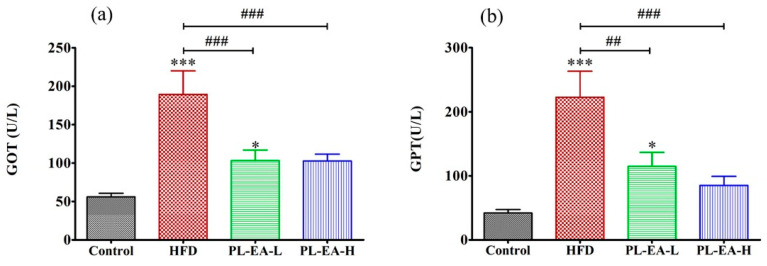
Effects of ethyl acetate fraction from *P. linteus* mycelia on the activities of (**a**) plasma aspartate aminotransferase and (**b**) plasma alanine aminotransferase. HFD: high-fat/high-fructose diet (HFD). PL-EA-L: HDF + low dose PL-EA (35 mg/kg). PL-EA-H: HDF + high dose PL-EA (70 mg/kg). Data are expressed as mean ± SE (*n* = 9). ## *p* <0.01; ### *p* < 0.001 vs. the HFD group; * *p* < 0.05; *** *p* < 0.001 vs. the control.

HFD apparently elevated the level of plasma alanine aminotransferase GOT to 185 ± 35 U/L compared to 56 ± 4 U/L of the control (*p* < 0.001), which markedly alleviated to 104 ± 15 U/L and 104 ± 10 U/L, respectively by PL-EA-L and PL-EA-H (*p* < 0.001) (Figure 5a). Similarly, the GPT level in the HFD mice rose to 225 ± 10 U/L, while PL-EA-L and PL-EA-H ameliorated the level to 155 ± 13 and 85 ± 8 U/L, respectively, compared to 40 ± 4 U/L of the control (Figure 5b). 

An increasing number of studies have demonstrated the promising hepatoprotective and antihepatotoxic effects of *P. linteus* [28,49,50]. Previously, Huang et al. [51], and recently Dong et al. [52], respectively, demonstrated the hepatoprotective bioactivity of hispidin [51] and hypholomine B [52]. Several investigations have proved the *Phellinus* species as being hepatoprotective and antihepatotoxic agents [36]. The compounds, phellinulin A [51], phellinulins D, E, F, G, H, I, K, M, and N, phenillin C, and γ-ionylideneacetic acid, when isolated from *P. linteus,* were all demonstrated to exhibit an hepatoprotective effect [51]. 

### 3.8. Oral Glucose Tolerance Test 

**Figure 6:**
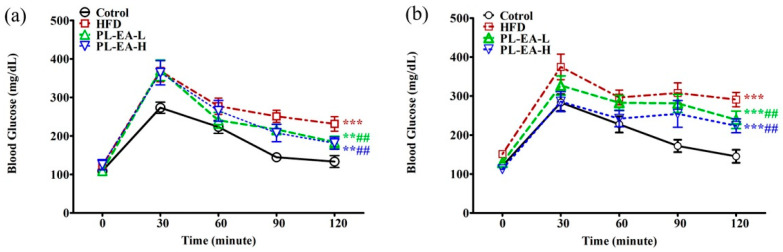
Effects of ethyl acetate fraction from *P. linteus* mycelia on the OGTT at week 9 (**a**) and at week 14 (**b**). HFD: high-fat/high-fructose diet (HFD). PL-EA-L: HDF + low dose PL-EA (35 mg/kg). PL-EA-H: HDF + high dose PL-EA (70 mg/kg). Data are expressed as mean ± SE (*n* = 9). ## *p* < 0.01; vs. the HFD group; ** *p* < 0.01; *** *p* < 0.001 vs. the control.

The plasma glucose level reached its peak value at 30 min in all groups after the tube feeding of glucose solution at week 9 (Figure 6a). Furthermore, HFD, PL-EA-H and PL-EA-L all comparably reached a plasma glucose level of 375 ± 20 mg/dL and a slight but insignificant deviation occurred between the three groups at 120 min compared to the control (135 ± 2 mg/dL) (*p* < 0.05) (Figure 6a). After treatment with PL-EA for 4 weeks, the plasma glucose profile changed at week 14 as follows: starting from 105–120 mg/dL at zero time, increasing to 385 ± 35 mg/dL (HFD), 325 ± 20 mg/dL (PL-EA-L), and 280 ± 16 mg/dL (PL-EA-H) at 30 min, declining steadily to 295 ± 14 mg/dL, 240 ± 17 mg/dL and 225 ± 15 mg/dL at 120 min, respectively, compared to 140 mg/dL for the control (Figure 6b). The results implicated the promising antihyperlipidemic and antihyperglycemic effects of PL-EA. However, it is advisable to slightly raise the dose of PL-EA for treatment instead.

Besides antioxidant activity, hispidin displays potentially hypoglycemic effects [53].

In chronic hyperglycemia, an excessive amount of glucose is shunted to the polyol pathway, where aldose reductase reduces glucose into sorbitol at the expense of NADPH. Since NADPH is essential for the generation of reduced glutathione (GSH, intracellular antioxidant) from oxidized glutathione (GSSG), the depletion of NADPH by the aldose reductase pathway may impair intracellular antioxidant defense [30]. Sorbitol can be converted to fructose via sorbitol dehydrogenase (SDH) with the production of NADH potentially leading to increased ROS via NADH oxidase [30]. 

Glucotoxicity may impair the regulation of glucokinase (GK) and its inhibitory protein, the GK regulatory protein (GKRP) [54], which plays a prognostic role in acute pancreatitis [55,56]. 

The fruiting body of *P. linteus* showed inhibitory activity against both the aldose reductase-related polyol pathway and protein glycation, effectively preventing artherosclerosis, cardiac dysfunction, retinopathy, neuropathy and nephropathy (Lee et al., 2008a; 2008b). The active principles of davallialactone, hypholomine B, and ellagic acid present in *P. linteus* exhibited potent human recombinant aldose reductase inhibitory activity [53]. 

### 3.9. HE Staining and Oil-Red Staining of Mice Liver Tissues

**Figure 7:**
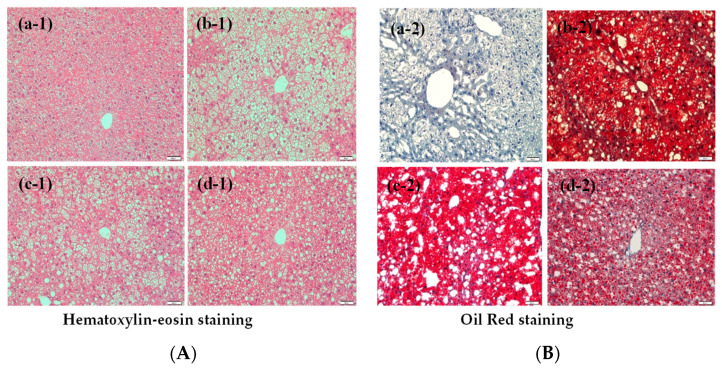
Effects of PL-EA on the hepatic lipogenesis in NAFLD mouse model. Representative photographs of hematoxylin-eosin (H&E) (**A**) and Oil Red O staining (**B**) of mice liver tissues for histological examination. (**a-1**,**a-2**) control. (**B**) (**b-1**,**b-2**) HFD, high-fat/high-fructose diet. (**c-1**,**c-2**) PL-EA-L: HDF + low dose PL-EA (35 mg/kg). (**d-1**,**d-2**) PL-EA-H: HDF + high dose PL-EA (70 mg/kg). Magnification, 200×.

To identify the effects of PL-EA on the expression of lipid accumulation in NAFLD mice, H&E staining and Oil-Red O staining of liver tissues were performed, respectively. HFD enhanced oil drop accumulation in the liver tissues (hepatic steatosis) (Figure 7(b-1,b-2)), compared to the control (Figure 7(a-1,a-2)). PL-EA dose dependently but incompletely alleviated such pathological changes (Figure 7(c-1,c-2,d-1,d-2)), suggesting a longer treatment time may be required. The HFD mice had a number of oil drops that accumulated in the tissues, most of which were reduced by feeding them PL-EA-L and PL-EA-H in a semi dose-dependent manner (Figure 7(c-1,c-2,d-1,d-2)). 

### 3.10. Effects of Purified Compounds from PL-EA on Free Fatty Acids-Induced Steatosis in HepG2 Cells

**Figure 8:**
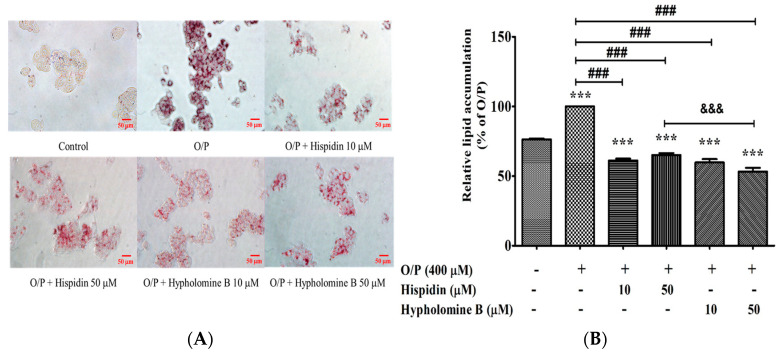
Effects of purified compounds from PL-EA on Oil Red O staining and lipid. accumulation in HepG2 cells. Lipid droplets in HepG2 cells were photographed by phase contrast microscopy (original magnification ×200) (**A**). The lipid content from Oil Red O stained cells was quantified by spectrophotometric analysis at 500 nm (**B**). Bars represent mean ± SE (*n* = 3) *** *p* < 0.001 vs. the control; ### *p* < 0.001 vs. the O/P group; ^&&&^ *p* < 0.001. Significant differences between groups were determined using one-way ANOVA followed by Tukey’s procedure.

The main active ingredients in the antioxidation of PL, such as hispidin and hypholomine B, have previously been reported [12]. However, it remains uncertain whether hispidin or hypholomine B are capable of antagonizing free fatty acids-induced hepatic steatosis. To investigate the effects of these two compounds on the lipid accumulation of hepatocytes, both hispidin and hypholomine B were purified from PL-EA using Hep G2 cell model. A total of 400 μM of the O/P treatment on HepG2 cells significantly induced lipid accumulation (24% increase at 24 h) compared with the control group (Figure 8A,B). Treatment with hispidin or hypholomine B (10 or 50 μM) and 400 μM of O/P significantly decreased lipid accumulation after 24 h of treatment (38% and 35% reduction with hispidin or 40% and 47% reduction with hypholomine B, at 10 and 50 μM, respectively). Furthermore, the contribution of the inhibitory activity of hypholomine B was significantly superior to that of hispidin at a concentration of 50 μM (Figure 8B). These findings suggest that the inhibition capability of hypholomine B is higher than that of hispidin, which is related to the ameliorative effect of oxidative stress according to a previous report [12].

### 3.11. Relative Gene Expression in Mice Liver Tissues 

**Figure 9:**
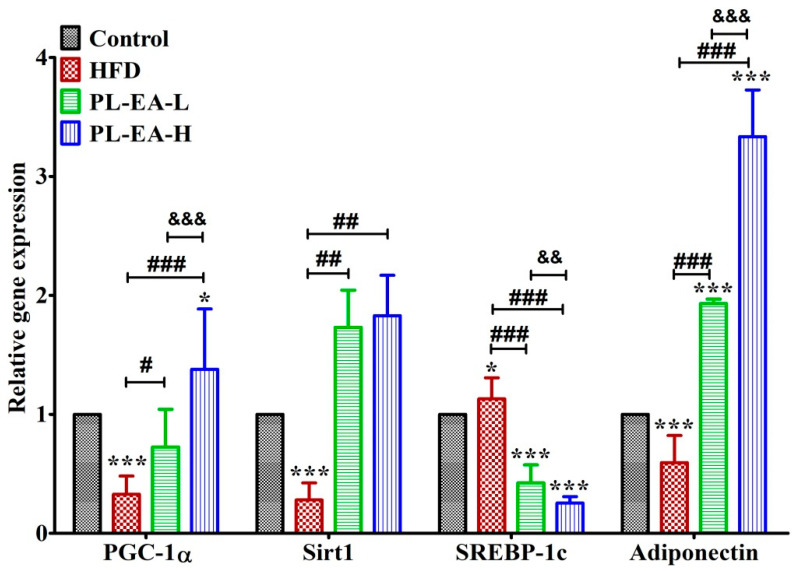
Effects of ethyl acetate fraction from *P*. *linteus* mycelia on the relative gene expression of mice livers. HFD: high-fat/high-fructose diet (HFD). PL-EA-L: HDF + low dose PL-EA (35 mg/kg). PL-EA-H: HDF + high dose PL-EA (70 mg/kg). Data are expressed as mean ± SE (*n* = 9). * and ***, *p* < 0.05 and 0.001, respectively vs. the control; ^#^, ^##^ and ^###^, *p* < 0.05, 0.01 and 0.001, respectively vs. the O/P group. ^&&^ and ^&&&^, *p* < 0.01 and 0.001, respectively, significant differences between the groups. PGC1-α: peroxisome proliferator-activated receptor gamma coactivator 1-alpha; Sirt-1: NAD-dependent deacetylase sirtuin-1; SREBP-1c: sterol regulatory element-binding protein 1c.

Some of the lipid-metabolism-related genes in the mice liver tissues were examined (Figure 9). As found, the genes PGC-1α, Sirt1, and adiponectin were all downregulated (*p* < 0.001), while SREBP-1c (*p* < 0.001) was upregulated with HFD. Feeding the mice with PL-EA apparently reversed such trends (Figure 9). Compared to that of HFD, the increments were as follows: for PGC-1α (+4.20 fold), Sirt1 (+6.53 fold), and adiponectin (+5.61 fold), following the administration of LP-EA-H (*p* < 0.001). In contrast, SREBP-1c was downregulated by 77.5% (*p* < 0.05) (Figure 9).

PGC-1α overexpression increased in markers of mitochondrial content and function; as a result, fatty acid oxidation was enhanced which was accompanied by reduced triacylglycerol accumulation and secretion [57]. 

SIRT1 is involved in both NAFLD and alcoholic fatty liver diseases (AFLD) [58]. An increased number of studies have provided evidence that SIRT1 acts as a key metabolic/energy sensor (via intracellular NAD^+^/NADH ratio) by transferring signals to initiate transcriptional activity and gene expressions that are involved in metabolic homeostasis [58,59,60].

Adiponectin has been revealed to protect the liver against hepatic steatosis by decreasing serum lipid and glucose production [61]. De novo lipogenesis has an important contribution to the pathophysiology of NAFLD because it provides almost one third of the accumulated hepatic triglycerides in patients with hepatosteatosis [52,62,63]. Thus, our findings suggest that PL-EA may improve hepatic steatosis and ameliorate HFD-induced fatty liver disease through the regulation of the hepatic fatty acids metabolism. 

### 3.12. Relative Gene Expression in HepG2 Cells

**Figure 10:**
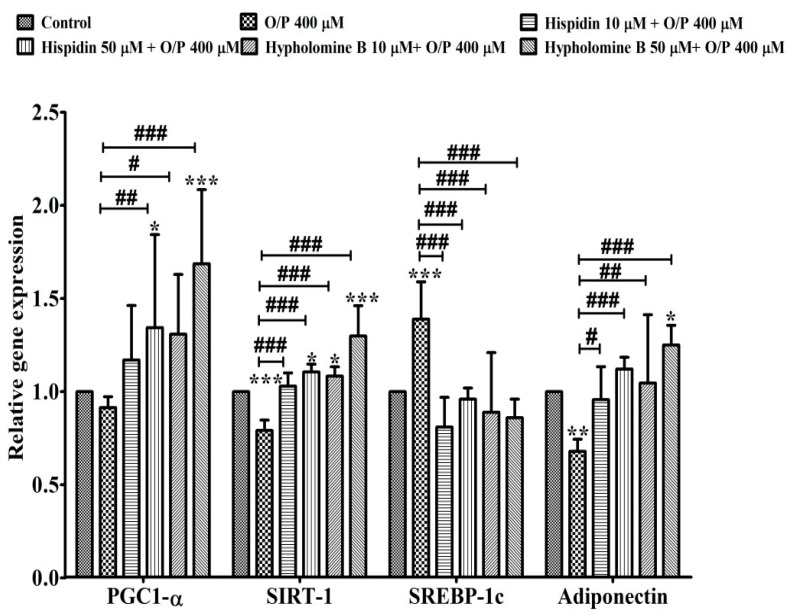
Effects of hispidin and hypholomine B isolated from the ethyl acetate fraction of *P*. *linteus* mycelia on the relative gene expression of HepG2 cell. Data are expressed as mean ± SE (*n* = 3). *, **, and ***, *p* < 0.05, 0.01 and 0.001, respectively vs. the control; ^#^, ^##^ and ^###^, *p* < 0.05, 0.01 and 0.001, respectively vs. the O/P group. PGC1-α: peroxisome proliferator-activated receptor gamma coactivator 1-alpha; Sirt-1: NAD-dependent deacetylase sirtuin-1; SREBP-1c: sterol regulatory element-binding protein 1c.

To further verify the possible candidates of active compounds in the PL-EA extract on NAFLD, the purified compounds of hispidin and hypholomine B were prepared and used in the genes expression analyses with an HepG2 cell model. After the exposure of HepG2 cells to O/P induction of a mimic steatosis, the cells were treated with hispidin or hypholomine for 24 h. Hypholomine B was found to show greater up-regulated activity in PGC1-α, SIRT1 and adiponectin genes expression than hispidin at the same dose of 50 μM (Figure 10). Nevertheless, the expression of PGC1-α, SIRT1 and adiponectin genes significantly increased in hispidin and hypholomine B at 10 and 50 μM, respectively, in comparison to the O/P group (Figure 10). Furthermore, expression of the lipogenesis-related gene SREBP-1c was elevated in HepG2 cells treated with 400 μM O/P. Hispidin or hypholomine B treatment significantly (*p* < 0.001) decreased the expression of the biogenesis marker (Figure 10). These results demonstrate that the main active ingredients of PL-EA may have pharmacological effects on NAFLD in both in vitro and in vivo studies. 

### 3.13. Western Blotting

**Figure 11:**
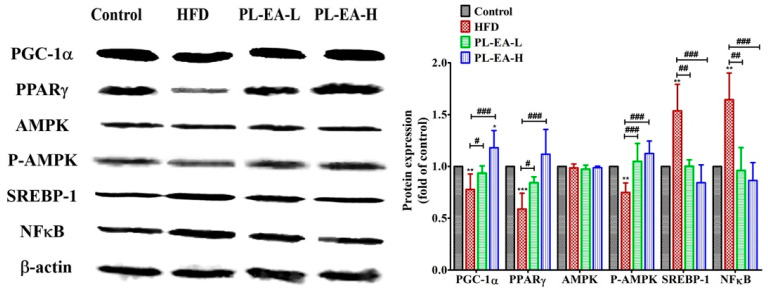
Effects of ethyl acetate fraction from *P. linteus* mycelia on the relative proteins expression of mice livers. Representative Western blots (left panel) and quantified bar graphs relative to each control (right panel) showing alterations among groups. HFD: high-fat/high-fructose diet (HFD). PL-EA-L: HDF + low dose PL-EA (35 mg/kg). PL-EA-H: HDF + high dose PL-EA (70 mg/kg). Data are expressed as mean ± SE (*n* = 9). # *p* < 0.05; ## *p* < 0.01; ### *p* < 0.001 vs. the HFD group; * *p* < 0.05; ** *p* < 0.01; *** *p* < 0.001 vs. control. PPARγ: peroxisome proliferator-activated receptor gamma. AMPK: AMP activated protein kinase. *p*-AMPK: phosphorylated AMPK. PGC1-α: peroxisome proliferator-activated receptor gamma coactivator 1-alpha. SREBP1: sterol regulatory element-binding protein 1. NF-κB: nuclear factor kappa-light-chain-enhancer of activated B cells.

HFD downregulated the expression of PPARγ, pAMPK, PGC1α, but upregulated SREBP-1 and NFκB in mice (Figure 11). PL-EA dose dependently alleviated the level of PPARγ and PGC1α. As for pAMPK, SREBP-1 and NFκB, both PL-EA-L and PL-EA-H showed very comparable effects. Compared to HDF, the PL-EA-L diet increased PPARγ 1.43 fold, p-AMPK 1.44 fold, PGC1α 1.19 fold. Conversely, it downregulated NFκB 52.5% and SREBP-1 35.8% (Figure 11). In contrast to that of PL-EA-L, PL-EA-H was found to be upregulated PPARγ 1.89 fold, p-AMPK 1.53 fold, and PGC1α 1.57 fold; it downregulated NFκB 57.5% and SREBP-1 45.2% (Figure 11).

PPARγ stimulates the expression of adiponectin and initiates signalling cascades in the liver, leading to increased β-oxidation, decreased gluconeogenesis and less insulin-resistant hepatic tissue [64]. PPARγ also induces phosphoenolpyruvate carboxykinase to facilitate the triglyceride synthesis [64], which obviously can be inhibited by PL-EA (Figure 4e). 

AMPK is a master regulator of the cellular metabolism and is responsible for the overall energy balance and the activation of AMPK (pAMPK) is recognized as an important regulator in the amelioration of NAFLD [65]. In addition, the upregulation of NFκB may reflect that the NAFLD is slightly associated with inflammation, which was apparently alleviated by PL-EA (Figure 11). 

**Figure 12:**
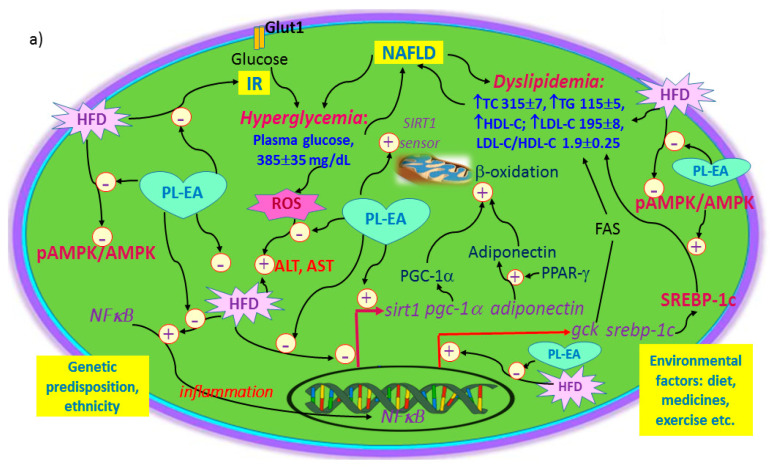

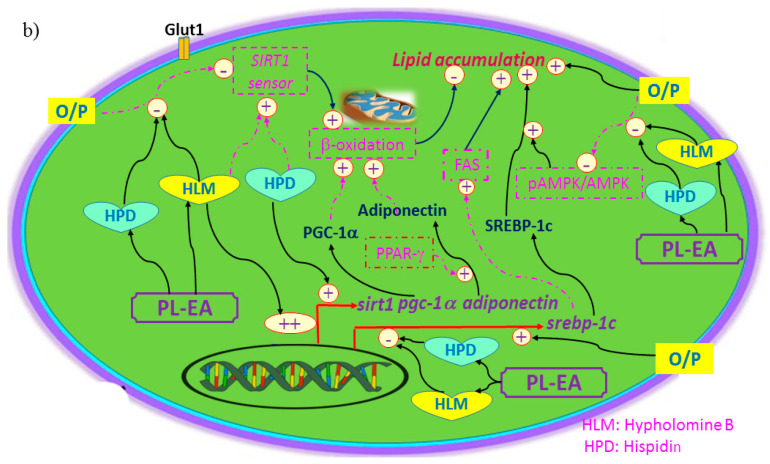
Mechanism of action related to the alleviative effect of NAFLD in mice with the ethyl acetate partition from *Phellinus linteus* (**a**) and that of in vitro hypolipidemic effect of hispidin and hypolomine B in HepG2 cell model (**b**). In Figure 12b, the items highlighted in pink are not shown in the HepG2 cell model but were found in the in vivo mice model. The purified active components from PL-EA used in the in vitro experiment are hispidin (HPD) and hypholomine B and isomers (HLM). ALT: alanine aminotransferase (GPT). AMPK: AMP-activated protein kinase. AST: aspartic aminotransferase (GOT). FAS: fatty acid synthase. *gck: glucokinase gene*. HFD: high fat diet. HDL-C: high density lipoprotein–cholesterol. IR: insulin resistance. LDL-C: low density lipoprotein–cholesterol. NAFLD: non-alcoholic fatty liver disease. NFκB: nuclear factor kappa-light-chain-enhancer of activated B cells. pAMPK: phosphorylated AMPK. *sirt-1*: NAD^+^-dependent deacetylase sirtuin-1 gene. PGC-1α: peroxisome proliferator-activated receptor-gamma coactivator-1α. *pgc-1α*: peroxisome proliferator-activated receptor-gamma coactivator-*1α gene*. PPAR-γ: peroxisome proliferator- activated receptor gamma (PPAR-γ). ROS: reactive oxygen species. SREBP-1c: sterol regulatory element-binding protein-1c. *srebp-1c*: sterol regulatory element-binding protein-1c gene. TC: plasma total cholesterol. TG: triglycerides. PL-EA: the ethyl acetate partition from *Phellinus linteus*.

To summarize, the underlying mechanisms of PL-EA for alleviating HFD-induced NAFLD are summarized in Figure 12a. Furthermore, the hypolipidemic effect with regard to the purified hispidin and hypholomine from the PL-EA is shown in Figure 12b.

## 4. Conclusions

Based on the presented results of this study, it could be concluded that 75% methanol extraction and solvent partition using ethyl acetate (PL-EA) is satisfactory for the extraction of bioactive antioxidants in PL mycelia. The pronounced antioxidant activity of PL-EA was associated with a high content of hispidin and, hypholomine B and its isomer. Furthermore, the results of the mouse model study indicate that the PL-EA has anti-NAFLD effects due to its regulation of hepatic lipogenesis and the potential antihyperglycemic effect it imparts. P. linteus has been traditionally utilized in East Asian countries for more than two hundred years and most of its active components have been evidenced elsewhere without toxic symptoms and complications. The implication of the study is that the development of a practically effective PL nutraceuticals therapy may be expected and demonstrated in the future.

## Figures and Tables

**Table 1:** The yield and total polyphenol content of *Phellinus linteus* mycelium freeze-dried powder from different extraction methods.

Extract	Yield (%)	Total Polyphenols (mg/g Freeze-Dried Mycelium)
Water	18.71 ± 0.31 ^b^	23.70 ± 6.09 ^c^
25% MeOH	23.68 ± 0.89 ^a^	21.22 ± 3.13 ^c^
50% MeOH	24.55 ± 1.64 ^a^	52.23 ± 4.58 ^b^
75% MeOH	21.93 ± 1.46 ^ab^	70.13 ± 5.90 ^a^
100% MeOH	19.83 ± 1.29 ^b^	47.42 ± 2.02 ^b^

Each value represents the mean ± SD of triplicate experiments. Values with different letters within the same column are significantly different (*p* < 0.05).

**Table 2:** Effects of ethyl acetate fraction from 75% methanol extracts of *Phellinus linteus* mycelium freeze-dried powder (PL-EA) on body weight; liver weight and liver to body weight ratio in a high-fat/high-fructose diet (HFD)-fed mouse model.

	Control	HFD	PL-EA-L	PL-EA-H
Body weight(g)				
Initial	21.32 ± 0.49	21.40 ± 0.81	21.10 ± 0.68	21.37 ± 0.94
Final	32.36 ± 1.71	40.00 ± 1.62 ***	35.83 ± 3.16 **^##^	35.50 ± 2.15 *^###^
Liver weight (g)	1.89 ± 0.25	3.22 ± 0.48 ***	2.49 ± 0.44 **^##^	2.31 ± 0.04 ^###^
Liver weight/ Body weight (%)	5.83 ± 0.62	8.06 ± 1.19 ***	7.02 ± 0.70 *^#^	6.48 ± 0.75 ^##^

HFD: high-fat/high-fructose diet, PL-EA-L: high-fat/high-fructose diet + low dose PL-EA (35 mg/kg b.w.), PL-EA-H: high-fat/high-fructose diet + high dose PL-EA (70 mg/kg b.w.). Data are expressed as means ± SE (*n* = 9). ^#^ *p* < 0.05; ^##^ *p* < 0.01; ^###^ *p* < 0.001 vs. the HFD group; * *p* < 0.05; ** *p* < 0.01; *** *p* < 0.001 vs. the control.

## Data Availability

All data generated or analyzed during this study are included in the published article (and its online Supplementary Files).

## Supplementary Materials

The following supporting information can be downloaded at: https://www.mdpi.com/article/10.3390/antiox11050898/s1, Table S1. Specific MRM settings for the styrylpyrone compounds from PL-EA and internal standard.; Table S2. List of primers for real-time PCR analyses in mouse liver and HepG2 cell; Table S3. Partition coefficients (K) of hispidin and hypholomine B in several different solvent systems; Figure S1. The total ion chromatogram from multiple reaction monitoring (top panel) and HPLC profile using diode array detection on ethyl acetate fraction of 75% methanol extract of Phellinus linteus mycelia.
