# Supplementary material for: Styrylpyrones from Phellinus linteus Mycelia Alleviate Non-Alcoholic Fatty Liver by Modulating Lipid and Glucose Metabolic Homeostasis in High-Fat and High-Fructose Diet-Fed Mice
Source: Antioxidants (Basel). 2022 Apr 30;11(5):898. doi: 10.3390/antiox11050898 (PMC9137645; doi:10.3390/antiox11050898)
Supplement: Supplementary file 1 [file antioxidants-11-00898-s001.zip › antioxidants-1711658-SI.pdf]

Supplementary materials

Styrylpyrones from *Phellinus linteus* Mycelia Alleviate Nonalcoholic Fatty Liver by Modulating Lipid and Glucose Metabolic Homeostasis in High-Fat and High-Fructose Diet-Fed Mice

Chun-Hung Chiu [1,2,†], Chun-Chao Chang [3,4,†], Jia-Jing Lin [1], Chin-Chu Chen [5], Charng-Cherng Chyau [1,*] and Robert Y. Peng [1,6,*]

[1] Research Institute of Biotechnology, Hungkuang University, Shalu District, Taichung City 43302, Taiwan; chchiu@hk.edu.tw (C.-H.C.); n404user@gmail.com (J.-J.L.)
[2] Department of Program in Animal Healthcare, Hungkuang University, Shalu District, Taichung City 43302, Taiwan
[3] Division of Gastroenterology and Hepatology, Department of Internal Medicine, Taipei Medical University Hospital, Taipei 11031, Taiwan; chunchao@tmu.edu.tw
[4] Division of Gastroenterology and Hepatology, Department of Internal Medicine, School of Medicine,
College of Medicine, Taipei Medical University, Taipei 11031, Taiwan
[5] Biotech Research Institute, GrapeKing Bio Ltd., Taoyuan 32542, Taiwan; gkbioeng@grapeking.com.tw
[6] Graduate Institute of Clinical Medicine, College of Medicine, Taipei Medical University, Taipei, Taiwan
* Correspondence: ccchyau@hk.edu.tw (C.-C.C.); ypeng@sunrise.hk.edu.tw (R.Y.P.); Tel.: +886-4-26318652 (C.-C.C.); Fax: +886-4-26525386 (C.-C.C.)
† These authors contributed equally to this work.

Table S1. Specific MRM settings for the styrylpyrone compounds from PL-EA and internal standard.

Peak No.	Compound	Quantitation transition (*m*/*z*)	Confirmation transition (*m*/*z*)	Fragmentor (V)	Collision energy (V)
1	Hispidin	159	201	115	15
2	Hispidin isomer	159	201	115	15
3	Hypholomine B	283	241	125	15
4	Hypholomine B isomer	283	241	125	15
5	IS*	151	107	135	20

*IS: internal standard, quercetin. PL-EA: Ethyl actate fraction of *Phellinus linteus* mycelia extract.

Table S2. List of primers for real-time PCR analyses in mouse liver and HepG2 cell.

Gene		Mouse liver Primers (5′ to 3′)	HepG2
β-actin	F	AAGACCTCTATGCCAACACAGT	CCCAGCACAATGAAGATCAAGATCAT
	R	AGCCAGAGCAGTAATCTCCTTC	ATCTGCTGGAAGGTGGACAGCGA
SIRT-1	F	CGTCTTGTCCTCTAGTTCCTGT	ACAGGTTGCGGGAATCCAAAGG
	R	GCCTCTCCGTATCATCTTCCA	CCTAGGACATCGAGGAACTACCTG
PGC-1α	F	TGATGTGAATGACTTGGATACAGACA	AGACACCGCACGCACCGAAAT
	R	GCTCATTGTTGTACTGGTTGGATATG	AGCTGTCATACCTGGGCCGACG
Adiponectin	F	GCTCTCCTGTTCCTCTTAATCC	AAGAAGCCATTATATACTCATAT
	R	ATGCCTGCCATCCAACCT	GGCACTGATAAGATCAATA
SREBP-1c	F	GATGTGCGAACTGGACACAG	CTCAACATGCACCCAGAAGA
	R	GCATGTCTTCGATGTCGTTCAAA	ACCATTGTGTTGCCTTCCTC

List of abbreviations: Sirt-1 = NAD-dependent deacetylase sirtuin-1； PGC1-α = Peroxisome proliferator-activated receptor gamma coactivator 1-alpha； SREBP-1c = sterol regulatory element-binding protein 1c; F: Forward, R: Reverse.

Table S3. Partition coefficients (K) of hispidin and hypholomine B in several different solvent systems.

**Two-phase solvent system**	**Ratio (v/v/v/v)**	**Hypholomine**		**Hispidin**	
		**Ka***	**Kd***	**Ka***	**Kd***
Petroleum ether- Ethyl acetate- Methanol-Water	0.8 : 1.8 : 1.0 : 2.1	1.86	0.58	2.61	0.38
Petroleum ether- Ethyl acetate- Methanol-Water	0.9 : 1.8 : 1.0 : 2.0	3.95	0.25	1.99	0.50
Petroleum ether- Ethyl acetate- Methanol-Water	1.6 : 2.3 : 1.0 : 2.1	1.46	0.68	1.12	0.89

* Ascending (Ka) and descending (Kd) modes, respectively.

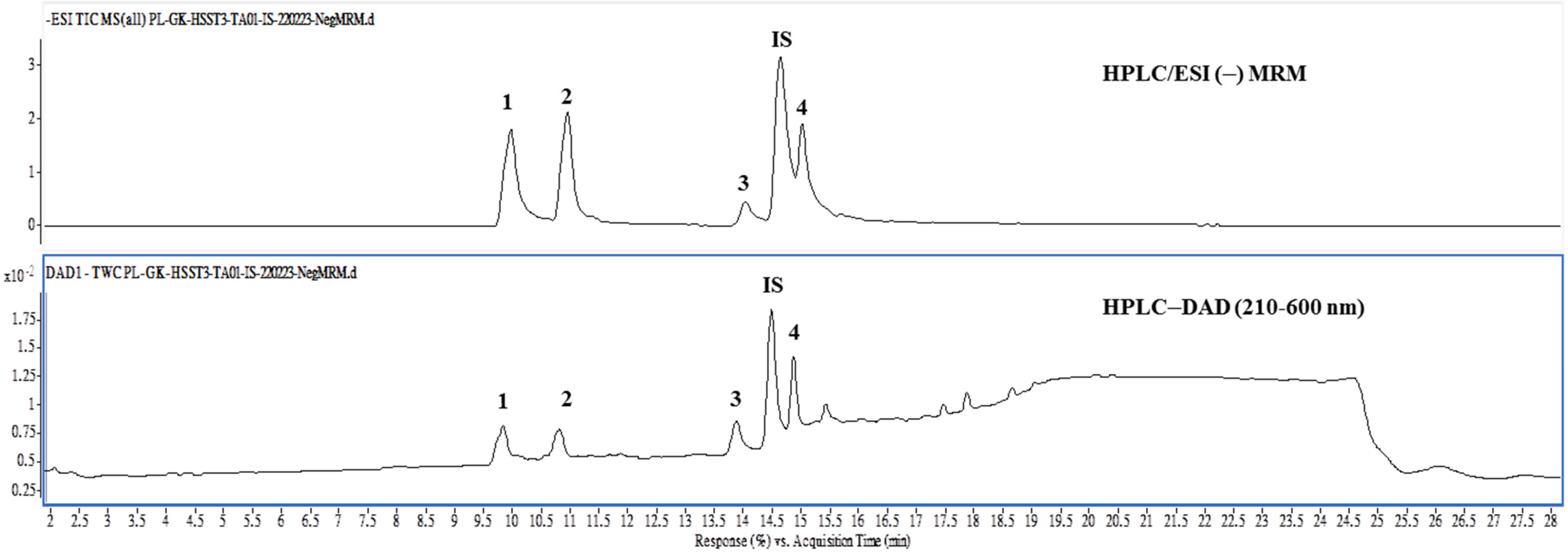

Figure S1. The total ion chromatogram from multiple reaction monitoring (top panel) and HPLC profile using diode array detection on ethyl acetate fraction of 75% methanol extract of *Phellinus linteus* mycelia. 1: hispidin, 2: hispidin derivative, 3: hypholomine B, 4: hypholomine B isomer and IS: internal standard quercetin.
